# COVID-19 exposure and depression-anxiety levels among Saudi adults in the Jazan region: a study with a predominantly female and undergraduate sample

**DOI:** 10.3389/fpsyt.2023.1200052

**Published:** 2023-08-14

**Authors:** Amani Busili, Azizah Makrami, Amnah Keni, Alaa Khardily, Dalyah Alzahrani, Idris Busaily, Fatimah Busayli

**Affiliations:** ^1^College of Medical and Dental Sciences, University of Birmingham, Birmingham, United Kingdom; ^2^Nursing College, Jazan University, Jazan, Saudi Arabia; ^3^Diabetes and Endocrinology Center, Jazan, Saudi Arabia; ^4^Damad General Hospital, Jazan, Saudi Arabia; ^5^Dental College, Jazan University, Jazan, Saudi Arabia; ^6^Sabya General Hospital, Jazan, Saudi Arabia

**Keywords:** COVID-19, anxiety, depression, COVID-19 exposure, Saudi Arabia (KSA)

## Abstract

**Background:**

The COVID-19 pandemic has caused significant mental health challenges worldwide, as evidenced by numerous studies indicating high levels of depression and anxiety among individuals. However, the extent of mental health disorders following the pandemic and the association between anxiety and depression and COVID-19 exposure levels in the Jazan region of the Kingdom of Saudi Arabia have received little research attention.

**Methods:**

A convenience sample of 377 participants, predominantly female (85.4%) with undergraduate education (74.5%) and Saudi nationality (92.8%), was included in the study. The study utilized a self-administered questionnaire to collect data from participants between 1st August and 8th September 2022. The questionnaire consisted of four parts, including demographic characteristics, COVID-19 exposure, the Patient Health Questionnaire-9 (PHQ-9) for depression assessment, and the Generalized Anxiety Disorders-7 (GAD-7) for anxiety evaluation. Statistical techniques such as descriptive statistics, independent *t*-tests, ANOVA (Analysis of Variance), and regression analysis were employed to analyze the collected data.

**Results:**

The mean age of the study participants was 30.97 years (SD = 9.072). The mean score for COVID-19 exposure was 2.98 (SD = 1.48). The mean level of depression was 7.83 (SD = 6.43), with 20% of participants experiencing moderate to severe depression. Additionally, the study found that the mean score of anxiety level among participants was 6.75 (SD = 6.57), with 26% of the participants experiencing moderate to severe anxiety. Independent *t*-test revealed significant differences in mean depression and anxiety scores between participants with varying COVID-19 exposure levels (*p* = 0.001). The regression analysis demonstrated that anxiety levels were significant predictors of depression (*p* < 0.001). There is a significant difference in the depression mean between participants with high levels of anxiety (≥10) compared to others with levels <10. Furthermore, significant predictors of anxiety levels included either student or unemployment status (*p* < 0.001), increased age (≥35) (*p* = 0.049), female gender (*p* = 0.009), marital status of not being married, divorced, or widowed (*p* = 0.004), low monthly income (*p* = 0.019), and increased depression level (*p* < 0.001).

**Conclusion:**

This study provides evidence of significant depression and anxiety levels among participants, with higher COVID-19 infection exposure correlating with increased scores for both. Anxiety was identified as a significant predictor of depression. Demographic factors, such as employment status, age, gender, and marital status, played a role in influencing anxiety levels. The findings highlight the need for targeted mental health interventions to address the psychological impact of COVID-19 infection exposure and support affected individuals effectively.

## Introduction

The global impact of the COVID-19 pandemic extended to Saudi Arabia, resulting in a significant health crisis (1, 2). Saudi Arabia has recorded a substantial number of confirmed COVID-19 cases and associated deaths (3). The COVID-19 pandemic has had a significant impact on psychological wellbeing globally, affecting both the general population and vulnerable subgroups. This has resulted in various symptoms of psychological distress, including fear, worry, avoidance, emotional symptoms, posttraumatic stress symptoms, substance abuse, physical symptoms, fatigue, loneliness, and aggressive behaviors (4–21).

It is inevitable that the COVID-19 has caused varying degrees of traumatization among almost all individuals (22). Extensive literature suggests that the magnitude of this impact is influenced by a complex interplay of biopsychosocial factors. For instance, individuals who demonstrate resilient stress responses and maintain a positive appraisal of the coronavirus crisis tend to exhibit more favorable psychological and biological outcomes during the pandemic (23, 24).

Furthermore, it has been recognized that COVID-19 itself can have significant implications for the mental wellbeing of those affected (25). Research utilizing electronic health records has investigated the correlation between neuropsychiatric symptoms and clinically diagnosed mental disorders (26, 27). A 1-year follow-up study using data from the US Veterans Affairs database examined the prevalence of mental disorders among 153,848 individuals who had survived SARS-CoV-2 infection. In comparison to both a contemporary and historical control group (27), the study found a prevalence of 1.35 for anxiety disorders and 1.39 for depressive disorders.

In the context of Saudi Arabia, a study conducted by Al-Gelban (28) highlighted anxiety, depression, and substance abuse as the most prevalent mental health conditions. Notably, the stigmatization of mental illness in Saudi society can create impediments to seeking mental health services, as individuals may fear the associated shame and discrimination (29). In a study by AlAteeq et al. (30), it was found that more than 50% of Saudi participants with a mood disorder reported concealing their mental illness from others to avoid situations that might subject them to stigmatization. However, COVID-19 may exacerbate the situation and increase the prevalence of mental health disorders. The Global Burden of Disease (GBD) study, conducted by Santomauro et al. (31), conducted a global analysis and estimated that the COVID-19 pandemic has resulted in a 28% increase [95% uncertainty interval (UI): 25–30] in major depressive disorders and a 26% increase (95% UI: 23–28) in anxiety disorders. These estimates were based on imputations and modeling using survey data on self-reported mental health problems.

The rapid and extensive spread of COVID-19 in Saudi Arabia (KSA) prompted individuals to implement a range of strategies and control measures, including welfare and relief initiatives, to address the escalating situation (1, 32). Additionally, research has indicated that the swift dissemination of COVID-19 within Saudi Arabia (KSA) has been associated with an upsurge in psychological symptoms among various segments of the population. These symptoms encompass stress, affective symptoms, insomnia, and obsessive-compulsive symptoms (33–42).

Nevertheless, the majority of the conducted studies in Saudi Arabia have taken place during the pandemic and lockdown periods, providing valuable insights into various aspects of the situation (33, 34, 36–45).

However, few studies have specifically investigated the association between mental health disorders and exposure to COVID-19 infection. This gap in the literature leaves us with a limited understanding of the prevalence of mental health disorders, particularly depression and anxiety, especially after discontinuing mandatory COVID-19 measures.

Addressing this gap is crucial from a clinical perspective. Gaining insights into the prevalence of depression and anxiety after the discontinuance of mandatory COVID-19 measures can guide healthcare providers and policymakers in developing targeted interventions and support systems. Understanding the potential relationship between these mental health disorders and exposure to COVID-19 infection can inform preventive measures and early interventions to mitigate the psychological impact of the pandemic.

By conducting this study, we aim to fill the existing knowledge gap and contribute to the body of evidence regarding mental health. The findings will not only enhance our understanding of the psychological consequences of the pandemic but also provide valuable insights for healthcare professionals to tailor their services and resources to meet the specific needs of individuals at high risk of depression and anxiety.

## Materials and methods

### Study design and sampling strategy

This cross-sectional study focused specifically on the Jazan region of Saudi Arabia to investigate the relationship between exposure to COVID-19 infection and depression-anxiety levels. The Jazan region, located at the southern border of the Kingdom of Saudi Arabia, has previously shown a seroprevalence of 26% for SARS-CoV-2 antibodies after the first wave of the pandemic (46). This emphasizes the importance of examining the association between mental health disorders, such as depression and anxiety, and COVID-19 exposure in this region.

To collect data, a convenience sampling method was utilized, resulting in a predominantly female sample (85.4%) with undergraduate education (74.5%). Social media platforms, including Facebook, WhatsApp, and Telegram, were employed as effective recruitment tools, leading to the completion of 377 online surveys.

The online survey consisted of mandatory questions to ensure participant responses and minimize response bias. The inclusion criteria were clearly defined, encompassing individuals aged 18 years and above, currently residing in Saudi Arabia, and without apparent cognitive deficiencies. Participants below 18 years of age or unable to understand the Arabic language were excluded.

All eligible participants were invited to voluntarily participate in the study and provided online informed consent. The survey was distributed between 1st August and 8th September 2022, after the discontinuation of various COVID-19 measures and policies, such as quarantine, lockdown, mandatory face mask usage, and social distancing guidelines in Saudi Arabia. However, certain recommendations remained in effect, advising individuals with COVID-19 symptoms to stay at home as a precautionary measure.

The sample size was determined through a formal power calculation using the Raosoft sample calculator software. The calculation was conducted at a 95% confidence level with a 5% confidence interval. The results showed that at least 377 participants are required for the study.

### Measures

The survey questionnaire include:

1. This study encompasses various demographic characteristics, including age, gender, family size, nationality, marital status, education level, monthly income, employment status, and presence of chronic diseases. The questionnaire comprises questions pertaining to these aspects.
2. The assessment of COVID-19 exposure involves five questions. Participants are asked about their own, their family members’, or friends’ diagnoses with COVID-19, their neighbors’ diagnoses with COVID-19, and if anyone in their household experienced symptoms of COVID-19 such as a high temperature or a dry cough or was suspected of having it. Each “yes” response to these questions was assigned one point. The total score ranges from 0 to 5, where a score of 3 or more is considered a high level of exposure.
3. The severity of depression was evaluated using The Patient Health Questionnaire-9 (PHQ-9) (47), a self-reported measure. The total scores were categorized as minimal or no depression (0–4), mild depression (5–9), moderate depression (10–14), moderately severe depression (15–19), or severe depression (20–27). The Arabic version of the PHQ-9 instrument, which had been validated among the Saudi population, was used. This version was obtained from the Saudi Ministry of Health (SMOH) website and was used with primary healthcare patients in primary healthcare centers. The PHQ-9 Arabic version showed good internal consistency, with a Cronbach’s alpha of 0.857. The test-retest reliability results showed that the PHQ-9 Arabic version was highly reproducible, with an ICC of 0.88 (0.71–0.95), *P*-value 0.001 (48). The cut-off score for depression was set at ≥10 points, as proposed by Kroenke et al. (47).
4. The severity of anxiety was assessed using The Generalized Anxiety Disorder (GAD-7) (49), a self-reported scale with high reliability and validity. The total scores were classified as minimal or no anxiety (0–4), mild anxiety (5–9), moderate anxiety (10–14), and severe anxiety (15–21). The Arabic version of the GAD-7, which had high validity, was used. This version was obtained from the SMOH website and was used with primary healthcare patients. The reliability of the Arabic version of the GAD-7 was acceptable, with a Cronbach’s alpha of 0.763 and all items were well correlated with the total scale as well as with each other. The cut-off score for anxiety was set at ≥10 points, as proposed by Spitzer et al. (49).

### Ethical consideration

In addition to obtaining ethical approval from the University of Jazan, this study also ensured the confidentiality of participant information. All data collected from participants was kept secure and only accessible to the research team.

Furthermore, the study adhered to principles of autonomy, meaning that participants were fully informed about the study’s purpose, procedures, and potential risks and benefits. They were given the opportunity to ask questions and provide online informed consent before participating. The consent form included information about the study’s confidentiality measures and reassured participants that their personal information would not be shared outside of the research team. Each participant had the option to withdraw from the study at any time without penalty.

### Statistical analysis

The collected data were analyzed using SPSS (Statistical Package for Social Sciences) version 26. Descriptive statistics were employed to summarize and describe the data, including the prevalence of mental health problems. Independent sample *t*-test were utilized to compare the means of two groups, and one-way ANOVA was employed for comparing means across more than two categorical groups, thereby enabling the assessment of significant differences in mental health among participants. All statistical analyses were conducted at a predetermined significance level of 0.05.

To explore the predictors of mental health disorders, a multiple regression analysis was performed using the Enter method. In this analysis, all independent variables, encompassing sociodemographic factors and COVID-19 exposure, were included as predictors. By examining the relationship between these variables and mental health disorders, the study sought to obtain a comprehensive understanding of the potential influences and associations between them.

## Results

### Demographic characteristics of participants

The study’s sample comprised 377 participants. Table 1 presents a summary of the participants’ demographic characteristics. The mean age was 30.97 (SD = 9.072), with a range of 18–62 years. Most of the participants were female (85.4%) and Saudi nationals (92.8%). Most were married (57.9%) and held an undergraduate degree (74.5%). In terms of monthly income, 34.5% earned more than 10,000 SAR^1^, while 33.4% earned less than 1,000 SAR. More than two-thirds (61%) had chronic diseases. In terms of employment status, more than half (51.5%) were employed, 46.1% were students, and 2.4% were not working.

**TABLE 1:** Demographic characteristics of participants (*N* = 377).

Characteristics	Mean	SD	Range
Age	30.97	9.072	18–62
Family size	5.47	3.231	1–20
Characteristics	Number	Percentage%	
Gender			
Female	322	85.4	
Male	55	14.6	
Nationality			
Saudi	350	92.8	
Non-Saudi	27	7.2	
Marital status			
Not married	129	34.2	
Married	218	57.8	
Divorced	21	5.6	
Widowed	9	2.4	
Education			
Primary	3	0.8	
Secondary	57	15.1	
Undergraduate	281	74.5	
Postgraduate	36	9.5	
Monthly income			
>1,000 SAR	126	33.4	
1,000–5,000 SAR	63	16.7	
5,000–10,000 SAR	58	15.4	
<10,000 SAR	130	34.5	
Chronic disease			
Yes	230	61	
No	147	39	
Employment status			
Student	174	46.1	
Employers	194	51.5	
Not employers	9	2.4	
COVID-19 exposure			
More exposure ≥3	277	73.5	
Less exposure <3	100	26.5	

### The level of COVID-19 exposure among study participants

The results of the study show that participants perceived a moderate to high level of COVID-19 exposure, with a mean score of 2.98 (SD = 1.48) and a median score of 3. Additionally, a considerable proportion of participants reported a personal or immediate family’s diagnosis of COVID-19, with 34.5% indicating that a family member had been diagnosed with the disease. Many participants (88.1%) reported knowing someone who had been diagnosed with COVID-19.

Regarding the participants’ communities, 70% reported that people in their community had been diagnosed with COVID-19. Moreover, 62.6% of the participants had someone living with them who had COVID-19 symptoms or was suspected of having the disease.

### Association between depression and independent variables among study participants: results of independent *t*-test and one-way ANOVA

The study found that the mean level of depression among participants was 7.83 (SD = 6.43), and 20% of the participants had moderate to severe depression using a cutoff score of 10 on the depression scale. Table 2 presents the means of independent variables in relation to depression levels based on independent *t*-Test and one-way ANOVA. The results indicated no significant differences in the mean scores between participants who were 35 years or older and those under 35 years (*p* = 0.709), or between males and females (*p* = 0.599). Additionally, the mean scores were not significant between those with a family size of less than five and those with a family size of five or more (*p* = 0.063), as well as between Saudi and non-Saudi individuals (*p* = 0.91) and between those infected with COVID-19 and those who were not (*p* = 0.805).

**TABLE 2:** Comparison of mean variables with depression levels based on independent *T*-test and one-way ANOVA.

Variables	Mean (SD)	*P*-value
Age		
≥35	7.60 (5.11)	0.709
<35	8.06 (7.11)	
Family size		
≥5	8.43 (6.97)	0.063
<5	7.20 (5.76)	
Gender		
Female	8.04 (6.88)	0.599
Male	7.16 (3.76)	
Nationality		
Saudi	7.70 (6.87)	0.91
Non-Saudi	9.85 (6.31)	
Marital status		
Married	7.27 (5.74)	0.385
Not married, divorced, or widowed	9.24 (7.95)	
Education		
Primary or secondary	10.79 (8.820)	0.002
Undergraduate	7.23 (6.02)	
Postgraduate	8.06 (3.77)	
Monthly income		
<1,000 SAR	8.31 (5.98)	0.021
1,000–5,000 SAR	8.52 (6.12)	
5,000–10,000 SAR	5.43 (4.38)	
>10,000 SAR	8.10 (7.51)	
Chronic disease		
No	6 (6.24)	0.008
Yes	9.14 (6.45)	
Employment status		
Student or not employers	9.04 (7.08)	0.001
Employers	7.06 (5.61)	
COVID-19 exposure		
More exposure ≥3	8.47 (6.33)	0.001
Less exposure <3	6 (6.40)	
COVID-19 infected		
No	7.09 (6.52)	0.805
Yes	7.43 (6.72)	
Anxiety level		
<10	5.89 (4.93)	0.001
≥10	13.35 (7.01)	

However, significant differences were observed in the mean scores between participants with different education levels (*p* = 0.002), monthly income levels (*p* = 0.021), chronic disease status (*p* = 0.008), employment status (*p* = 0.001), and COVID-19 exposure levels (*p* = 0.001).

Furthermore, participants with an anxiety level less than 10 had a lower mean score of depression, while those with an anxiety level greater than or equal to 10 had a significantly higher mean score. The difference in means between the two groups was found to be statistically significant with a significance level of 0.001.

### Association between anxiety and independent variables among study participants: results of independent *t*-test and one-way ANOVA

The study found that the mean score of anxiety level among participants was 6.75 (SD = 6.57), and 26% of the participants had moderate to severe anxiety using a cutoff score of 10 on the anxiety scale. Table 3 displays the mean scores and standard deviations of different independent variables in relation to anxiety levels among participants using one-way ANOVA and independent sample *t*-test. The findings revealed that age, family size, gender, marital status, nationality, and COVID-19 infection did not show significant differences (*p* > 0.05). However, education, monthly income, chronic disease, employment status, COVID-19 exposure, and depression levels showed significant differences among participants (*p* < 0.05).

**TABLE 3:** Comparison of mean variables with anxiety levels based on independent *T*-test and one-way ANOVA.

Variables	Mean (SD)	*P*-value
Age		
≥35	7.47 (7.15)	0.474
<35	6.54 (6.57)	
Family size		
≥5	6.87 (7.13)	0.715
<5	6.62 (5.94)	
Gender		
Female	7.20 (7.03)	0.140
Male	4.66 (4.14)	
Nationality		
Saudi	7.14 (6.87)	0.007
Non-Saudi	3.42 (2.87)	
Marital status		
Married	6.42 (6.44)	0.178
Not married, divorced, or widowed	8.58 (7.45)	
Education		
Primary or secondary	9.37 (8.15)	0.011
Undergraduate	6.31 (6.40)	
Postgraduate	6.25 (3.98)	
Monthly income		
<1,000 SAR	9.40 (7.31)	0.001
1,000-4,999 SAR	5.83 (570)	
5,000–10,000 SAR	3.24 (2.94)	
>10,000 SAR	6.19 (6.44)	
Chronic disease		
No	3.53 (4.21)	0.001
Yes	8.94 (7.22)	
Employment status		
Student or not employers	9.21 (7.75)	0.001
Employers	4.86 (4.37)	
COVID-19 exposure		
More exposure ≥3	7.78 (6.92)	0.001
Less exposure <3	4.68 (4.91)	
COVID-19 Infected		
No	6.09 (6.67)	0.168
Yes	4.24 (4.27)	
Depression level		
<10	5.39 (5.79)	0.001
≥10	12.23 (6.70)	

The multiple linear regression analysis presented in Table 4 examined the predictors of depression levels. The results indicate that among the variables assessed, anxiety level emerged as a statistically significant predictor of depression (β = 0.626, *p* < 0.001). These findings suggest a significant positive relationship between anxiety level and depression, indicating that as anxiety level increases, depression levels are expected to increase accordingly.

**TABLE 4:** Multiple linear regression analysis results for depression.

Variables	*B*	Std. error	Beta	*T*	*P*-value
Anxiety level	0.606	0.076	0.626	7.970	<0.001
Employment status	0.088	1.193	0.001	0.006	0.995
Age	−0.075	0.059	−0.104	−1.287	0.201
Gender	0.916	1.502	0.049	0.610	0.543
Marital status	0.419	0.887	0.046	0.473	0.634
Monthly income	0.816	0.478	0.159	1.706	0.091
Education	−1.179	0.984	−0.095	−1.198	0.233
Family size	0.119	0.183	0.058	0.650	0.517
Nationality	−0.758	1.829	−0.031	−0.414	0.680
COVID-19 exposure	0.408	0.345	0.094	1.182	0.240

Table 5 displays the results of a multiple linear regression analysis aimed at examining the relationship between several independent variables and anxiety level. The analysis yielded some significant predictors of anxiety level, including employment status (*p* < 0.001), age (*p* = 0.049), gender (*p* = 0.009), marital status (*p* = 0.004), monthly income (*p* = 0.019), and depression level (*p* < 0.001).

**TABLE 5:** Multiple linear regression analysis results for anxiety.

Variables	*B*	Std. error	Beta	*T*	*P*-value
Employment status	−2.506	0.632	−0.207	−3.969	<0.001
Age	0.061	0.031	0.085	1.975	0.049
Sex	−2.109	0.797	−0.113	−2.645	0.009
Marital status	−1.361	0.471	−0.147	−2.891	0.004
Monthly income	−0.615	0.262	−0.119	−2.351	0.019
Education	−0.032	0.536	−0.003	−0.059	0.953
Family size	−0.030	0.096	−0.015	−0.308	0.758
Nationality	1.173	0.985	0.047	1.190	0.235
COVID-19 exposure	0.133	0.188	0.030	0.706	0.481
Depression level	0.577	0.041	0.564	14.122	<0.001

^1^Saudi Arabian Riyal.

These results indicate that individuals who are students or unemployed, older, female, unmarried (single, divorced, or widowed), or have lower monthly income are more likely to experience higher levels of anxiety.

Conversely, some independent variables were found to have no significant relationship with anxiety level, including education (*p* = 0.953), family size (*p* = 0.758), nationality (*p* = 0.235), and COVID-19 exposure (*p* = 0.481).

## Discussion

This study aimed to assess the prevalence of mental health disorders, specifically depression and anxiety, and to investigate the relationship between these disorders and exposure to COVID-19 infection.

The study revealed that the mean level of depression among participants was 7.83 (SD = 6.43), with 20% of the participants experiencing moderate to severe depression, as determined by a cutoff score of 10 on the depression scale. Furthermore, the study found that the mean score of anxiety level among participants was 6.75 (SD = 6.57), with 26% of the participants experiencing moderate to severe anxiety, using a cutoff score of 10 on the anxiety scale.

Participants who had higher levels of exposure to COVID-19 were found to have significantly higher levels of depression and anxiety than those with less exposure. The results of the regression analysis further emphasized the significance of certain factors in predicting depression level. Specifically, the findings suggest that anxiety level is a significant predictor of depression level (*t* = 7.970, *p* < 0.001). Moreover, there is a significant difference in the mean depression scores between participants with high levels of anxiety (≥10) compared to those with lower levels (<10).

Furthermore, significant predictors of anxiety levels included either student or unemployment status (*p* < 0.001), increased age (≥35) (*p* = 0.049), female gender (*p* = 0.009), marital status of not being married, divorced, or widowed (*p* = 0.004), low monthly income (*p* = 0.019), and increased depression level (*p* < 0.001).

The study’s results indicate a significant burden of mental health disorders in the population. The findings are particularly concerning as 20% of participants experienced moderate to severe depression and 26% displayed symptoms of anxiety. These findings align with previous studies, suggesting that the pandemic has had a lasting impact on mental health (50–52). The high prevalence of depression and anxiety is consistent with other studies conducted during the pandemic, which suggest that the pandemic has led to a significant increase in mental health disorders (53, 54). The findings highlight the need for continued monitoring and support for mental health services in the aftermath of the pandemic, as the impact of the pandemic on mental health may persist even after the pandemic has subsided (55, 56).

When comparing the results based on previous studies conducted on the general population in Saudi Arabia, this study’s findings revealed that 20% of the participants experienced moderate to severe depression, and 26% experienced severe or extremely severe anxiety. These rates were relatively lower than those reported in previous studies conducted among various population categories in Saudi Arabia, such as healthcare workers (57, 58). It is important to note that these studies primarily focused on healthcare workers, who are known to be particularly affected by pandemics and natural disasters due to their direct exposure (58). However, the prevalence reported in this study is lower compared to a systematic review study conducted by Alzahrani et al. (43), in which the overall prevalence was 30% for depression and 29% for anxiety. This inconsistency may be attributed to the studies included in the systematic review, which were conducted during a year of COVID-19 pandemic characterized by uncertainty, unanticipated, unemployment, and mandatory isolation, all of which caused adverse psychological effects.

In contrast, our study demonstrated a higher prevalence of depression and anxiety symptoms compared to a cross-sectional study conducted among the general Saudi population. The earlier study reported a rate of 13.9% experiencing severe symptoms of anxiety and 16.4% experiencing severe symptoms of depression, even though more than half of the participants were female and had at least a bachelor’s degree (39). Several factors could contribute to the discrepancy in these findings. Firstly, the timing of data collection in our study occurred after a significant number of participants had been infected, which could have influenced the higher prevalence of depression and anxiety symptoms. Additionally, variations in the scales and cut-off points used for psychological assessment might have influenced the outcomes. Therefore, to obtain a comprehensive understanding of the psychological impact of the pandemic and accurately determine prevalence rates over time, a longitudinal study is warranted. Such a study would allow for a more in-depth analysis of changes in depression and anxiety levels and provide valuable insights into mental health care.

In addition, our results are higher compared to the national study conducted by Alhabeeb et al. (59) using a cross-sectional design. They employed a phone interview survey with 6,015 participants, utilizing a quota sampling strategy. In their study, the national prevalence of individuals at risk of Major Depressive Disorder (MDD) and Generalized Anxiety Disorder (GAD) was found to be 12.7 and 12.4%, respectively. These disparities in prevalence may be attributed to the overrepresentation of chronic disease patients and female participants in our study. However, it is important to note that the national study did not specifically report the percentage of people with chronic diseases. Therefore, comparing the two studies directly may have some limitations.

Study results demonstrated a significant difference in the levels of depression and anxiety between participants who had high exposure to the COVID-19 infection and those who had low exposure to the infection. A recent study conducted by Alhakami et al. (36) in Saudi Arabia also supports these findings, revealing increased levels of COVID-19 anxiety syndrome in individuals diagnosed with COVID-19 compared to those without a diagnosis. This suggests that the COVID-19 anxiety syndrome may contribute to the long-lasting psychological symptoms associated with stressful events related to COVID-19 (14, 60–62). Furthermore, the COVID-19 exposure has been found to increase the use of dysfunctional coping strategies, such as avoidance behaviors (61, 63), due to the fear and threat associated with the virus. These maladaptive coping mechanisms can trap individuals in a perpetual state of fear and anxiety, hindering their recovery and normal functioning. Consequently, this sustained distress may contribute to the persistence of psychological disorders beyond the pandemic’s duration (61, 64). Moreover, a COVID-19 diagnosis can trigger various forms of anxiety, such as persistent concerns about the virus, excessive worry about contracting it, and preoccupation with associated bodily symptoms (7, 8, 65–68).

However, it is important to note that a study conducted by Mansueto et al. (14) in Italy found no significant difference in the occurrence of COVID-19 anxiety syndrome between individuals who had been exposed to COVID-19 and those who had not. These disparities in findings may be attributed to methodological variations between the studies, including cultural, social, and healthcare differences. To gain a comprehensive understanding of the relationship between COVID-19 infection, anxiety, and depression across diverse populations and contexts, further research is necessary.

Another possible explanation for the significant association between COVID-19 infection exposure and higher levels of depression and anxiety among participants could be attributed to biological factors. In a study conducted by Kucukkarapinar et al. (69), the researchers investigated the potential link between alterations in the tryptophan-kynurenine (TKP) pathway and the occurrence of psychiatric symptoms following COVID-19 infection. The findings of the study suggested that changes in the TKP pathway might contribute to the development of long-term psychiatric disorders, specifically depression and anxiety, after exposure to COVID-19. These results highlight the potential of the TKP pathway as a biomarker for identifying these psychiatric disorders, and targeting this pathway could have implications for preventing future viral infections associated with depression and anxiety. Therefore, it is crucial to conduct comprehensive studies utilizing a biopsychosocial model to enhance our understanding of the complex relationship between the biological effects of the virus on individuals exposed to COVID-19 infection and the psychological aspects of the pandemic, including the fear of infection and the impact of isolation. By considering biological, psychological, and social factors, such research would provide valuable insights into the broader implications of COVID-19 on mental health. Furthermore, it would facilitate the development of targeted interventions and support systems that address the unique needs of individuals affected by the COVID-19 infection and the ongoing challenges posed by the pandemic.

The finding that anxiety level is a significant predictor of depression level in our study is in line with previous research that has shown a strong association between these two mental health issues (70). This suggests that addressing anxiety may be a crucial step in preventing or treating depression. Additionally, our study found a trend toward significance for monthly income as a predictor of depression level, indicating that financial stressors may also contribute to the development of depression. These results highlight the importance of considering both mental health and financial factors when addressing the impact of the pandemic on mental health.

The results of our study suggest that several demographic and mental health factors may play a role in the development of anxiety. Specifically, our findings indicate that unemployment status, increased age, female gender, marital status (single, divorced, or widowed), low monthly income, and increased depression levels are all significant predictors of anxiety levels.

These findings are consistent with previous research that has identified similar demographic and mental health factors as predictors of anxiety in general [e.g., (36, 39, 44, 71, 72)]. For example, previous studies have suggested that individuals unemployed or low income are more likely to experience anxiety than those employed or have higher income (44). Similarly, research has shown that women are more likely to experience anxiety than men (36, 39, 44, 71). Previous evidence has indicated that females were approximately two times more likely than men to experience symptoms of depression, anxiety, and stress during the pandemic (39). There are several potential reasons for the heightened probability of anxiety among women. Even though many COVID-19 measures have been discontinued, concerns and fears regarding infection remain, particularly among women (14, 36). For example, the high level of COVID-19 anxiety syndrome in women compared with men could potentially contribute to a significant prevalence of mental health disorders among women (14, 36).

Several studies conducted in Saudi Arabia have reported similar findings to our study regarding the strong association between depression and anxiety during the pandemic. For example, a study by AlAteeq et al. (73) found that individuals with higher levels of depression were more likely to experience anxiety symptoms during the pandemic. These studies support our findings and suggest that addressing both depression and anxiety may be crucial for promoting mental health.

### Limitation

Several limitations should be taken into consideration when interpreting the findings of this study. Firstly, the study focused on the exposure to COVID-19 infection without considering the timing of the infection. The timing of infection could potentially influence the level of mental health disorders experienced by individuals. Additionally, the exposure is dependent on self-reported results provided by the participants, without determining evidence of infection such as a positive COVID-19 test or other diagnostic tests. Additionally, other important factors associated with the pandemic, such as isolation, lockdown measures, mask wearing in public locations, and working from home, were not included in the analysis. These factors have been recognized as significant contributors to mental health outcomes during the pandemic and their exclusion may limit the comprehensive understanding of the impact on mental wellbeing.

Secondly, the use of convenience sampling in this study introduces a potential limitation. Convenience sampling involves selecting participants based on their accessibility and willingness to participate, often using readily available individuals or groups (74). While convenience sampling can be convenient and cost-effective, it may result in a biased sample that does not accurately represent the broader population of interest. In this study, participants were recruited primarily through online platforms and social media, which may attract individuals who are more active online or have specific characteristics that differ from the general population.

Furthermore, there was an overrepresentation of females and undergraduates in the study, which may have skewed the findings and limited their generalizability. The higher proportion of females and individuals enrolled in undergraduate programmes in the study may not adequately capture the experiences and mental health outcomes of other demographic groups within the Saudi Arabian population, such as males, or individuals with different educational backgrounds. This overrepresentation of certain groups restricts the ability to draw comprehensive conclusions about the entire population.

Indeed, an important limitation of our study is the potential influence of confounding factors on the reported prevalence of mental health disorders. Participants who had greater exposure to the COVID-19 pandemic and individuals with pre-existing chronic diseases could introduce biases that may affect the findings. It is crucial to recognize that individuals with higher exposure to the pandemic might experience heightened levels of depression and anxiety. Similarly, participants with pre-existing chronic diseases may have a higher baseline risk for mental health disorders, potentially leading to an overestimation of prevalence compared to the general population.

Additionally, it is important to consider the potential for response bias due to the use of self-administered surveys. Self-report measures of mental health may be influenced by social desirability bias, where participants provide responses that they perceive as socially acceptable rather than accurately reflecting their true experiences. This could affect the validity and reliability of the collected data. To obtain more accurate outcomes, it is essential to conduct both retrospective and prospective studies. Retrospective studies involve examining past events or data to analyze their effects on mental health outcomes. On the other hand, prospective studies involve following participants over time to observe and assess changes in mental health. By conducting both types of studies, researchers can gather more precise and reliable data, enabling them to reinforce the need for targeted public mental health strategies with stronger evidence. This comprehensive approach would enhance our understanding and inform effective interventions in mental health promotion.

Furthermore, one notable limitation of this study is the utilization of a small sample size. The number of participants included in the research was limited, which may have implications for the generalizability and statistical significance of the results. Therefore, conducting research with larger and more diverse samples could provide more comprehensive insights and enhance the reliability of the study’s findings.

Lastly, it is important to note that the cross-sectional design of this study introduces a limitation in terms of establishing causal relationships (75). A cross-sectional research approach captures data at a single point in time, providing a snapshot of mental health without accounting for the sequence of events or the long-term effects of the pandemic. Therefore, drawing definitive conclusions about cause and effect becomes challenging. Therefore, future research could consider employing a longitudinal design, which would allow for the examination of changes over time and provide more insights into the long-term impacts of the pandemic on mental health.

## Conclusion

The study identified a substantial burden of mental health disorders, specifically depression and anxiety, among the participants. It was observed that individuals with higher exposure to COVID-19 infection experienced significantly elevated levels of depression and anxiety. Given these findings, it is crucial to address both depression and anxiety to promote mental wellbeing in Saudi Arabia. Sustained monitoring and provision of support for mental health services are necessary in the post-pandemic period, as the lingering effects on mental health may persist even after the pandemic has subsided.

## Data availability statement

The raw data supporting the conclusions of this article will be made available by the authors, without undue reservation.

## Ethics statement

The studies involving humans were approved by the Review Ethics Committees at Jazan University, Saudi Arabia. The studies were conducted in accordance with the local legislation and institutional requirements. The participants provided their written informed consent to participate in this study.

## Author contributions

AB was responsible for drafting the initial manuscript. AM, DA, AmK, and AlK contributed to the data visualization and oversaw project supervision and administration and ensuring adherence to ethical and scientific standards. IB and FB took charge of revising the manuscript, addressing reviewers’ comments, and proofreading the final version. All authors made significant contributions to the study’s conception, design, data acquisition, analysis, and interpretation, provided the critical feedback and revisions to ensure its accuracy and clarity, and thoroughly reviewed and approved the final version of the manuscript.
